# Supplementary material for: Contribution of telacebec to novel drug regimens in a murine tuberculosis model
Source: Antimicrob Agents Chemother. 2024 Dec 9;69(1):e00962-24. doi: 10.1128/aac.00962-24 (PMC11838007; doi:10.1128/aac.00962-24)
Supplement: Supplemental material — Tables S1 to S6. [file aac.00962-24-s0001.docx]

**Supplementary Material**

**Table S1a. Experimental scheme Experiment 1 (H37Rv)**

|  | **Time point and # of mice** | | | | |
| --- | --- | --- | --- | --- | --- |
| **Regimen** | **W-2** | **D0** | **W4** | **W8** | **Total** |
| Untreated | 2 | 3 |  |  | **5** |
| Pa_50_L_100_ |  |  |  | 4 | **4** |
| B_25_PaL |  |  |  | 4 | **4** |
| T_5_PaL |  |  |  | 4 | **4** |
| PaM_100_Z_150_ |  |  | 4 |  | **4** |
| BPaMZ |  |  | 4 |  | **4** |
| TPaMZ |  |  | 4 |  | **4** |
| MZRb_5_ |  |  | 4 |  | **4** |
| BMZRb |  |  | 4 |  | **4** |
| TMZRb |  |  | 4 |  | **4** |
| BMZ |  |  | 4 |  | **4** |
| BMZT |  |  | 4 |  | **4** |
| PMZ |  |  | 4 |  | **4** |
| PMZT |  |  | 4 |  | **4** |
| 286_35_A_200_ |  |  |  | 4 | **4** |
| B286A |  |  |  | 4 | **4** |
| T286A |  |  |  | 4 | **4** |
| TC_6.25_286A |  |  |  | 4 | **4** |
| **Total** | **2** | **3** | **40** | **28** | **73** |

Number in subscript indicate dose in mg/kg. *Untreated mice were euthanized at week 3.

Abbreviations: Pa=pretomanid; L=linezolid; B=bedaquiline; T=telacebec; M=moxifloxacin, Z=pyrazinamide; Rb=rifabutin; 286=GSK-286; A=TBA-7371; C=clofazimine. W-2 = Day after infection; D0 = Day of treatment initiation; W4 and W8 = 4 and 8 weeks after treatment initiation, respectively.

Dosing was once daily 5 days a week.

**Table S1b. Mean CFU counts at week 4 and week 8 in Experiment 1 (H37Rv)**

|  | **Time point and mean (±SD) log_10_ CFU counts** | | | |
| --- | --- | --- | --- | --- |
| **Regimen** | **W-2** | **D0** | **W4** | **W8** |
| Untreated | 4.61 ± 0.19 | 7.69 ± 0.33 |  |  |
| Pa_50_L_100_ |  |  |  | 4.81 ± 0.46 |
| B_25_PaL |  |  |  | 1.13 ± 0.24 |
| T_5_PaL |  |  |  | 5.86 ± 0.24 |
| PaM_100_Z_150_ |  |  | 6.59 ± 0.22 |  |
| BPaMZ |  |  | 4.51 ± 0.41 |  |
| TPaMZ |  |  | 5.91 ± 0.36 |  |
| MZRb_5_ |  |  | 5.75 ± 0.15 |  |
| BMZRb |  |  | 2.78 ± 0.40 |  |
| TMZRb |  |  | 4.53 ± 0.16 |  |
| BMZ |  |  | 2.38 ± 0.05 |  |
| BMZT |  |  | 2.64 ± 0.33 |  |
| PMZ |  |  | 4.97 ± 0.62 |  |
| PMZT |  |  | 4.78 ± 0.13 |  |
| 286_35_A_200_ |  |  |  | 6.84 ± 0.15 |
| B286A |  |  |  | 1.44 ± 0.17 |
| T286A |  |  |  | 7.14 ± 0.32 |
| TC_6.25_286A |  |  |  | 3.70 ± 0.83 |

Number in subscript indicate dose in mg/kg.

Abbreviations: Pa=pretomanid; L=linezolid; B=bedaquiline; T=telacebec; M=moxifloxacin, Z=pyrazinamide; Rb=rifabutin; 286=GSK-286; A=TBA-7371; C=clofazimine.

W-2 = Day after infection; D0 = Day of treatment initiation; W4/8 = 4/8 weeks after treatment initiation, respectively.

**Table S2a. Experimental scheme Experiment 2 (H37Rv)**

|  | **Time point and # of mice** | | | |
| --- | --- | --- | --- | --- |
| **Regimen** | **W-2** | **D0** | **W4** | **Total** |
| Untreated | 2 | 3 |  | **5** |
| S876_6.25_Pa_30bid_L_25bid_ |  |  | 5 | **5** |
| S876_6.25_ |  |  | 5 | **5** |
| S876C_6.25_ |  |  | 5 | **5** |
| S876CT_5_ |  |  | 5 | **5** |
| S876CT+L_25bid_ |  |  | 5 | **5** |
| S876CT+U_25bid_ |  |  | 5 | **5** |
| S876CT+O_45bid_ |  |  | 5 | **5** |
| S876CT+Rb_5_ |  |  | 5 | **5** |
| S876CT+Pa_30bid_ |  |  | 5 | **5** |
| S876CT+Mp_50_ |  |  | 5 | **5** |
| S876CT+286_35_ |  |  | 5 | **5** |
| S876CT+A_75bid_ |  |  | 5 | **5** |
| S876CT+Z_150_ |  |  | 5 | **5** |
| **Total** | **2** | **3** | **65** | **70** |

Number in subscript indicate mg/kg/dose.

Abbreviations: S876=TBAJ-876; C=Clofazimine; T=telacebec; L=Linezolid; U=Sutezolid; O=TBI-223; Rb=Rifabutin; Pa=Pretomanid; Mp=MPL-446; 286=GSK-286; A=TBA-7371; Z=Pyrazinamide. W-2 = Day after infection; D0 = Day of treatment initiation; W4 = 4 weeks after treatment initiation.

Dosing was once daily except for L, U, O, Pa, and A which were dosed twice daily, all regimens were given 5 days a week.

**Table S2b. Mean CFU counts at week 4 in Experiment 2 (H37Rv)**

|  | **Time point and mean (±SD) log_10_ CFU counts** | | |
| --- | --- | --- | --- |
| **Regimen** | **W-2** | **D0** | **W4** |
| Untreated | 4.14 ± 0.12 | 7.51 ± 0.06 |  |
| S876_6.25_Pa_30bid_L_25bid_ |  |  | 3.96 ± 0.22 |
| S876_6.25_ |  |  | 3.74 ± 0.44 |
| S876C_6.25_ |  |  | 1.88 ± 0.28 |
| S876CT_5_ |  |  | 2.65 ± 0.19 |
| S876CT+L_25bid_ |  |  | 3.03 ± 0.27 |
| S876CT+U_25bid_ |  |  | 2.84 ± 0.20 |
| S876CT+O_45bid_ |  |  | 2.51 ± 0.50 |
| S876CT+Rb_5_ |  |  | 2.51 ± 0.23 |
| S876CT+Pa_30bid_ |  |  | 4.18 ± 0.15 |
| S876CT+Mp_50_ |  |  | 3.26 ± 0.21 |
| S876CT+286_35_ |  |  | 2.11 ± 0.51 |
| S876CT+A_75bid_ |  |  | 2.44 ± 0.64 |
| S876CT+Z_150_ |  |  | 0.00 ± 0.00* |

Number in subscript indicate mg/kg/dose.

* 1.66 log_10_ was the lower limited of detection in this study

Abbreviations: S876=TBAJ-876; C=clofazimine; T=telacebec; L=linezolid; U=sutezolid; O=TBI-223; Rb=rifabutin; Pa=pretomanid; Mp=MPL-446; 286=GSK-286; A=TBA-7371; Z=pyrazinamide.

Dosing was once daily except for L, U, O, Pa, and A which were dosed twice daily, all regimens were given 5 days a week.

W-2 = Day after infection; D0 = Day of treatment initiation; W4 = 4 weeks after treatment initiation.

**Table S3a. Experimental scheme Experiment 3 (H37Rv)**

|  | **Time point and # of mice** | | | | |
| --- | --- | --- | --- | --- | --- |
| **Regimen** | **W-2** | **D0** | **W4** | **W8** | **Total** |
| Untreated | 2 | 4 | 4* |  | **10** |
| T_10_ |  |  | 4 |  | **4** |
| T_50_ |  |  | 4 |  | **4** |
| S587_12.5_ |  |  | 4 |  | **4** |
| T_10_ + S587 |  |  | 4 | 4 | **8** |
| T_50_ + S587 |  |  | 4 | 4 | **8** |
| S587C_12.5_ |  |  | 4 | 4 | **8** |
| S587Z_150_ |  |  | 4 |  | **4** |
| T_10_ + S587C |  |  | 4 | 4 | **8** |
| T_10_ + S587Z |  |  | 4 |  | **4** |
| T_10_ + C |  |  | 4 | 4 | **8** |
| T_10_ + Z |  |  | 4 | 4 | **8** |
| **Total** | **2** | **4** | **48** | **24** | **78** |

*Untreated mice were euthanized at week 3.

Number in subscript indicate dose in mg/kg.

Abbreviations: S587=TBAJ-587; T=telacebec; C=clofazimine; Z=pyrazinamide; W-2 = Day after infection; D0 = Day of treatment initiation; W4 and W8 = 4 and 8 weeks after treatment initiation, respectively.

Dosing was once daily 5 days a week.

**Table S3b. Mean CFU counts at week 4 and week 8 in Experiment 3 (H37Rv)**

|  | **Time point and mean (±SD) log_10_ CFU counts** | | | | |
| --- | --- | --- | --- | --- | --- |
| **Regimen** | **W-2** | **D0** | **W3*** | **W4** | **W8** |
| Untreated | 4.41 ± 0.02 | 7.52 ± 0.13 | 9.41 ± 0.23 |  |  |
| T_10_ |  |  |  | 7.65 ± 0.05 |  |
| T_50_ |  |  |  | 7.70 ± 0.06 |  |
| S587_12.5_ |  |  |  | 3.40 ± 0.47 |  |
| T_10_ + S587 |  |  |  | 4.40 ± 0.17 | 2.33 ± 0.08 |
| T_50_ + S587 |  |  |  | 4.54 ± 0.14 | 2.27 ± 0.23 |
| S587C_12.5_ |  |  |  | 2.57 ± 0.28 | 0.00 ± 0.00 |
| S587Z_150_ |  |  |  | 2.20 ± 0.37 |  |
| T_10_ + S587C |  |  |  | 3.15 ± 0.22 | 0.78 ± 0.00 |
| T_10_ + S587Z |  |  |  | 2.74 ± 0.20 |  |
| T_10_ + C |  |  |  | 6.05 ± 0.27 | 3.53 ± 0.23 |
| T_10_ + Z |  |  |  | 4.53 ± 0.25 | 2.92 ± 0.37 |

Number in subscript indicate dose in mg/kg. *untreated mice were euthanized at week 3.

Abbreviations: S587=TBAJ-587; T=telacebec; C=clofazimine; Z=pyrazinamide.

W-2 = Day after infection; D0 = Day of treatment initiation; W3/4/8 = 3/4/8 weeks after treatment initiation, respectively.

**Table S4a. Mean concentrations of S587 at steady state**

|  | | **Regimen** | | | |
| --- | --- | --- | --- | --- | --- |
|  |  | **S587**_12.5_ | **S587+T_10_** | **S587+T_50_** | **S587+T**_10_**C**_12.5_ |
| **Time-point** | **1h** | 794±323 | 465±115 | 331±373 | 93±57 |
|  | **5h** | 411±151 | 242±146 | 424±216 | 120±62 |
|  | **24h** | 310±158 | 264±165 | 279±206 | 75±16 |

Concentration of S587 in ng/ml ± SD. Number in subscript indicate mg/kg/dose.

Abbreviations: S587=TBAJ-587; T=telacebec; C=clofazimine

**Table S4b. Mean concentrations of M3 metabolite of S587 at steady state**

|  | | **Regimen** | | | |
| --- | --- | --- | --- | --- | --- |
|  |  | **S587**_12.5_ | **S587+T_10_** | **S587+T_50_** | **S587+T**_10_**C**_12.5_ |
| **Time-point** | **1h** | 1160±387 | 1259±348 | 1127±1305 | 292±208 |
|  | **5h** | 1601±311 | 811±602 | 2696±219 | 497±96 |
|  | **24h** | 909±378 | 906±527 | 1358±501 | 194±41 |

Concentration of metabolite 3 of S587 in ng/ml ± SD. Number in subscript indicate mg/kg/dose.

Abbreviations: S587=TBAJ-587; T=telacebec; C=clofazimine

**Table S4c. Mean concentrations of T at steady state**

|  | | **Regimen** | | | | |
| --- | --- | --- | --- | --- | --- | --- |
|  |  | **T_10_** | **S587**_12.5_**+T_10_** | **S587+T_10_C**_12.5_ | **T_50_** | **S587+T_50_** |
| **Time-point** | **1h** | 1462±435 | 1585±509 | 1445±1056 | 2642±1857 | 4608±6536 |
|  | **5h** | 2265±102 | 861±644 | 1843±379 | 7310±3990 | 9881±2812 |
|  | **24h** | 921±337 | 722±477 | 670±113 | 3008±330 | 2297±886 |

Concentration of Telacebec in ng/ml ± SD. Number in subscript indicate mg/kg/dose.

Abbreviations: S587=TBAJ-587; T=telacebec; C=clofazimine

**Table S5a. Experimental scheme for Experiment 4 (HN878)**

|  | **Time point and # of mice** | | | |
| --- | --- | --- | --- | --- |
| **Regimen** | **W-2** | **D0** | **W4** | **Total** |
| Untreated | 2 | 3 |  | **5** |
| B_25_C_6.25_ |  |  | 4 | **4** |
| BCT_5_ |  |  | 4 | **4** |
| BCM_100_ |  |  | 4 | **4** |
| BCMT |  |  | 4 | **4** |
| Pa_50_L_100_ |  |  | 4 | **4** |
| BPaL |  |  | 4 | **4** |
| BPaLT |  |  | 4 | **4** |
| TPaL |  |  | 4 | **4** |
| BPaC |  |  | 4 | **4** |
| BPaCT |  |  | 4 | **4** |
| BPaM |  |  | 4 | **4** |
| BPaMT |  |  | 4 | **4** |
| BMZ_150_ |  |  | 4 | **4** |
| BMZT |  |  | 4 | **4** |
| B286_35_A_200_ |  |  | 4 | **4** |
| B286AT |  |  | 4 | **4** |
| PaRb_5_ |  |  | 4 | **4** |
| PaRbT |  |  | 4 | **4** |
| P_10_MZ |  |  | 4 | **4** |
| PMZT |  |  | 4 | **4** |
| **Total** | **2** | **3** | **80** | **85** |

Number in subscript indicate mg/kg/dose.

Abbreviations: B=Bedaquiline; C=Clofazimine; T=telacebec; M=Moxifloxacin; Pa=Pretomanid; L=Linezolid; Z=Pyrazinamide; A=TBA-7371; Rb=Rifabutin. W-2 = Day after infection; D0 = Day of treatment initiation; W4 = 4 weeks after treatment initiation.

Dosing was once daily 5 days a week.

**Table S5b. Mean CFU counts at week 4 in Experiment 4 (HN878)**

|  | **Time point and mean (±SD) log_10_ CFU counts** | | |
| --- | --- | --- | --- |
| **Regimen** | **W-2** | **D0** | **W4** |
| Untreated | 4.13 ± 0.04 | 6.73 ± 0.10 |  |
| B_25_C_6.25_ |  |  | 3.12 ± 0.36 |
| BCT_5_ |  |  | 0.41 ± 0.70 |
| BCM_100_ |  |  | 0.00 ± 0.00 |
| BCMT |  |  | 0.00 ± 0.00 |
| Pa_50_L_100_ |  |  | 6.31 ± 0.23 |
| BPaL |  |  | 4.04 ± 0.21 |
| BPaLT |  |  | 3.43 ± 0.49 |
| TPaL |  |  | 6.05 ± 0.21 |
| BPaC |  |  | 2.50 ± 0.28 |
| BPaCT |  |  | 0.39 ± 0.45 |
| BPaM |  |  | 2.90 ± 0.12 |
| BPaMT |  |  | 3.17 ± 0.16 |
| BMZ_150_ |  |  | 0.20 ± 0.39 |
| BMZT |  |  | 0.94 ± 0.66 |
| B286_35_A_200_ |  |  | 0.00 ± 0.00 |
| B286AT |  |  | 0.57 ± 0.66 |
| PaRb_5_ |  |  | 6.38 ± 0.32 |
| PaRbT |  |  | 6.00 ± 0.20 |
| P_10_MZ |  |  | 5.96 ± 0.23 |
| PMZT |  |  | 4.77 ± 0.12 |

Number in subscript indicate mg/kg/dose.

Abbreviations: B=bedaquiline; C=clofazimine; T=telacebec; M=moxifloxacin; Pa=pretomanid; L=linezolid; Z=pyrazinamide; 286=GSK’286; A=TBA-7371; Rb=rifabutin; P=rifapentine.

W-2 = Day after infection; D0 = Day of treatment initiation; W4 = 4 weeks after treatment initiation.

**Table S6. MICs of antitubercular drugs used in this study against *M. tuberculosis* H37Rv and HN878**

|  | **MIC (μg/ml)** | | | |
| --- | --- | --- | --- | --- |
| **Drug** | **7H9 Broth** | | **7H11 Agar** | |
|  | H37Rv | HN878 | H37Rv | HN878 |
| Telacebec |  |  | 0.008 | 0.002 |
| Bedaquiline | 0.25 | 0.125 | 0.03 | 0.03 |
| TBAJ-876 |  |  | 0.002-0.004 | 0.004 |
| TBAJ-587 |  |  | 0.008 | 0.004 |
| Clofazimine |  |  | 0.25 | 0.25 |
| Pretomanid | 0.125 | 0.06 | 0.06 | 0.125 |
| Linezolid^1^ | 1 | 0.5 | 0.5 | 0.5 |
| Moxifloxacin | 0.5 | 0.25 |  |  |
| Rifampin | 0.25 | 0.25 |  |  |
| TBA-7371 |  |  | 0.25 | 0.5 |
| Pyrazinamide* | <100 | <100 |  |  |
|  |  |  |  |  |
| *Pyrazinamide susceptibility determined by MGIT | | | | |
